# The Effect of Blurred Perceptual Training on the Decision Making of Skilled Football Referees

**DOI:** 10.3389/fpsyg.2018.01803

**Published:** 2018-09-27

**Authors:** Tammie van Biemen, J. Koedijker, Peter G. Renden, David L. Mann

**Affiliations:** ^1^Department of Human Movement Sciences, Amsterdam Movement Sciences and Institute for Brain and Behavior Amsterdam, Vrije Universiteit Amsterdam, Amsterdam, Netherlands; ^2^Faculty of Health, Nutrition & Sport, The Hague University of Applied Sciences, The Hague, Netherlands

**Keywords:** perceptual training, decision making, referee, football, blur

## Abstract

When judging ambiguous foul situations in football (soccer), referees must attune to the kinematic characteristics inherent in genuine fouls to ensure that they can (i) recognize when a foul has taken place, and (ii) discriminate the presence of deceptive intent on the part of the tackled player. The aim of this study was to determine whether perceptual training that removes superficial visual information would improve the decision-making performance of football referees. Two groups of skilled referees judged ambiguous foul situations on video before and after a training intervention that involved adjudicating foul situations. During the training phase, participants in a blurred-footage training group watched digitally altered, blurred videos that removed superficial visual information, whilst participants in a normal-footage control group viewed the same videos without blur (i.e., with the superficial information present). We hypothesized that blurred-training would train referees to ignore superficial visual information and instead focus on the basic kinematic movements that would better reveal the true nature of the inter-personal interaction. Consistent with this idea, training with blurred footage resulted in a positive change in response accuracy from pre to post-test when compared with normal-footage training. This improvement could not be explained on the basis of changes in response time or bias, but instead reflected a change in the sensitivity to genuine fouls. These findings provide a promising indication of the potential efficacy of blurred-footage training for referees to attune to the kinematic information that characterizes a foul. Blurred training might offer an innovative means of enhancing the decision-making performance of football referees via perceptual training.

## Introduction

Football (soccer) referees who adjudicate high-level professional matches are faced with an extraordinarily difficult task. They make an average of 137 decisions about goals, free-kicks, and penalties per 90-min match (Helsen and Bultynck, 2004), all while being scrutinized by players, spectators, and pundits at the match, and potentially by millions of fans watching at home. The most common, and probably most critical decision that referees are faced with are those in which they must differentiate whether a foul has been committed by one player on their opponent, or instead in some cases whether the opponent has taken a “dive” in an attempt to fool the referee into awarding an unjustified foul (Helsen and Bultynck, 2004). In these ambiguous foul situations, the referee is required to “see through” any deceptive intent on the part of the tackled player to judge whether a genuine foul has taken place. The consequences of an incorrect decision can be significant, particularly if a foul is awarded in the team’s penalty area, with a penalty shot often resulting in a goal being scored. Data from Top-4 leagues in England show that the outcome of ≈60% of football games are decided by a maximum of one goal difference between the teams (data from top-4 leagues in England; Curley, 2016), making correct decisions vital, and training approaches which minimize errors when adjudicating ambiguous foul situations are clearly desirable (Schweizer et al., 2011; Pizzera and Raab, 2012a).

There are a variety of social contexts in which it is important to be able to perceive deceptive intent (Cañal-Bruland, 2017). Much of the work on deception has its origins in verbal interactions, whereby one person may wish to determine whether another is lying (Ekman et al., 1999; Vrij and Mann, 2004). Research on deception has also been extended to understanding physical interactions, where an observer may seek to anticipate the movement intentions of others. There are a variety of situations in which a person may wish to produce movements that deceive others (e.g., pickpockets, magicians), and this is particularly the case in sports where an advantage can be gained by forcing opponents into misjudging action outcomes, such as one–on–one interactions in rugby, tennis and football. Evidence shows that expert athletes are not only better able to produce deceptive actions, but they also possess a better capability to “see through” this deceptive intent to more accurately anticipate the true action intentions of their opponents (Jackson et al., 2006).

It is not only skilled athletes who possess superiority in their ability to anticipate the deceptive actions of others; skilled sports officials also are better able to discriminate deceptive from non-deceptive actions. For instance, Renden et al. (2014) have shown that football referees have an advantage in their ability to distinguish genuine from deceptive actions when making judgements about ambiguous foul situations in football. In their study, Renden et al. (2014) recruited skilled football referees to make judgements of ambiguous foul situations seen in video footage from actual matches, and compared the judgements of the referees to those of skilled players, wheelchair-bound football fans, and novices. Results revealed that the referees and players outperformed the fans and novices, demonstrating that both groups are better able to discriminate genuine from deceptive fouls in football. The superior performance of the players provided some support for the idea that motor experience through playing the game may have contributed to the superior performance of the players. However, the concurrent superiority of the referees suggested that their perceptual experience in viewing and making decisions in ambiguous foul scenarios was sufficient to support success. This raises the possibility that the visual experience gained via additional perceptual training, which supplements the amount of exposure to these situations, may help to further improve the decision-making ability of referees [see also Luis del Campo et al. (2018)].

The ability to anticipate the outcome of a motor action is underpinned by an ability to interpret the kinematic movements producing that action, particularly when the action contains deceptive intent. Runeson and Frykholm (1983) first demonstrated the role of kinematics in deception. In their experiment, Runeson and Frykholm showed observers recorded videos of an actor lifting a box onto a table. In the videos, the actor was shown as a point-light representation so that the image of the actor was replaced by points of light at each if their key joint centers. Observers were very good at performing the perceptual task, even when the actor attempted to deceive them by pretending that the box was heavier than it really was. Because observers could detect this deception when viewing a point-light representation of the actor, the results suggest that the genuine action intentions are revealed via the basic kinematic movements of the actor. More recently, Abernethy et al. (2010a,b) compared the anticipatory skill of badminton players when watching both videos and point-light displays of an opponent playing deceptive and non-deceptive badminton strokes. The results revealed that deception was effective when players observed the videos of the opponent, but was less effective when viewing the point-light displays. In the point-light displays, observers were better able to see through the deceptive intent of the opponent. On the basis of these findings it was reasoned that deceptive intent must be conveyed largely via superficial (non-kinematic) visual information such as contour, color, and texture that is present in the video, but is not present in point-light display. Genuine intent on the other hand, is largely conveyed through basic kinematic information that is present in the point-light displays. In support, the quality of decisions made when making judgements about moving stimuli has been shown in some situations to improve when superficial visual information is removed via the use of visual blur (Di Lollo and Woods, 1981; Luria and Newacheck, 1992), and in particular when blur is applied while anticipating the actions of others (Jackson et al., 2009; Mann et al., 2010; Ryu et al., 2015, 2016). This suggests that it would be beneficial to learn to ignore superficial (non-kinematic) information when seeking to improve the anticipation of a deceptive movements, and instead to attune to the basic kinematic signature that specifies the movement outcome.

Ryu et al. (2018) have recently demonstrated that perceptual training using a blurred rather than clear image may be advantageous when making judgements about movement outcomes in the presence of deceptive intent. In their study, novice badminton players trained to anticipate the actions of an opponent when viewing video footage that displayed (i) blurred information only, (ii) highly detailed information only, or (iii) normal video footage. When tested following training, the results revealed that those who trained with blurred video footage experienced the greatest improvement in their ability to predict the outcome of deceptive badminton strokes. It was reasoned that the blurred-training group may have better learned to ignore the superficial bodily information, and instead attuned to the underlying kinematic pattern that they had viewed during training, that being the information which best specifies the genuine action outcome of the opponent.

While deception may be specified in many cases by non-kinematic information, there is reason to believe that kinematic information may have an important role to play during ambiguous foul situations when conveying deceptive intent. Morris and Lewis (2010) performed a simple observational comparison of football tackles in which attempts at deception (i.e., dives) clearly were and were not present. Morris and Lewis concluded that the two sets of tackles could be distinguished by differences in kinematics, with diving often characterized by: (i) the presence of the “archer’s bow,” a form of diving characterized by arms raised and backward, chest thrust out and legs bent at the knees; (ii) a discontinuity between the moment of contact and the supposed effect on the tackled player; (iii) an exaggeration in the effect of the force on the tackled player; and (iv) by spatial misalignment between where contact was made and where the tackled player implied that contact took place. Given that the deceptive intent reported in Morris and Lewis’s study was very obvious on the basis of the kinematic differences, the authors speculated that tackled players used highly exaggerated kinematic actions to ensure that observers (including referees) could clearly see the substantial effect of the tackle. This clearly noticeable behavior may be necessary given that the referee often stands a substantial distance away from the incident. Nonetheless, the findings highlight that referees must be attuned to the kinematic characteristics inherent in genuine fouls to ensure that they can (i) recognize when a foul has taken place, and (ii) discriminate the presence of deceptive intent on the part of the tackled player.

The aim of this study was to determine whether perceptual training that removes superficial visual information would improve the decision-making performance of skilled football referees. To do so, skilled referees were allocated to one of two training groups: a blurred-footage training group who adjudicated foul situations when watching video clips that were blurred; and a normal-footage (control) training group who trained viewing the same videos without blur. Based on previous research which has shown the efficacy of perceptual training for improving performance in refereeing (Catteeuw et al., 2010; Schweizer et al., 2011; Pizzera and Raab, 2012a; Put et al., 2013, 2016a,b), we expected both training groups to improve their decision-making accuracy following training. Crucially, we hypothesized that the addition of blur during training would better attune observers to the kinematic information that specifies the genuine movement outcome, resulting in a greater improvement in pre vs. post-test performance for the blurred-footage training group when compared to the normal-footage control group.

## Materials and Methods

### Participants

A total of 22 skilled male referees (*M_age_ ± SD* = 31.3 ± 8.1 yrs) from the Dutch National Football Association (KNVB) took part in the experiment (*M_experience_ ± SD* = 13.4 ± 5.5 yrs). The referees where either professional or semi-professional, refereeing international (*N* = 4) and/or national in the three highest national Dutch soccer leagues (“Eredivisie,” “Jupiler League,” and “Tweede divisie”). All referees had adjudicated matches at the national level for at least 1 year (*M ± SD* = 6.9 ± 4.6 yrs) and were unfamiliar with video-based perceptual training. They had normal or corrected-to-normal vision. Participants were randomly assigned to one of two training groups (with the allocated group alternating in order of participation): a blurred-footage training group who trained viewing blurred stimuli (*n* = 11; *M_age_ ± SD* = 30.7 ± 7.3 yrs; *M_experience_* ±*SD* = 13.4 ± 5.6 yrs; three internationals), or a normal-footage control group, who trained viewing standard (non-manipulated) video stimuli (*n* = 11; *M_age_* ±*SD* = 31.9 ± 9.1 yrs; *M_experience_* ±*SD* = 13.5 ± 5.7 yrs; one international). Participants provided written informed consent to a procedure that conformed to the Declaration of Helsinki and was approved by the Vrije Universiteit Amsterdam Faculty of Human Movement Sciences Ethics committee (approval number VCWE-2016-212).

### Study Design

A short-term training study was conducted using a pre-post test design. For practical reasons related to the restricted availability of the referees, training and testing were all conducted on the same day, with the entire procedure taking approximately 45 min for each participant.

### Procedure

During the pre-test, participants were asked to judge potential foul situations as a “foul” or “no foul” when viewing video clips displayed on a laptop computer (HP ZBook15, 15.6 inch, 1920 × 1080 pixels). For testing, we used the same clips as Renden et al. (2014), with situations taken from the 2006 FIFA World Cup. Correct responses had been judged by an expert panel of two experienced (Dutch accredited) soccer referees [for details, see original paper of (Renden et al., 2014)]. Participants took part in three practice trials before commencing the pre-test. Following the method of Renden et al., the test consisted of a total of 26 clips: 13 showing fouls and 13 showing no foul. The 50:50 split of fouls vs. no fouls was used to minimize the potential influence of any pre-conceived priors that the referees might have had about the likelihood of a foul taking place. Video clips were presented in a different random order for each participant using OpenSesame software (Mathôt et al., 2012). Each clip commenced with the trial number shown on the screen, followed by a countdown from 3 to 1 to cue the commencement of the clip. Participants were required to decide as quickly and accurately as possible whether the incident seen in the clip should be judged as a “foul” or “no foul” and to press a corresponding key on the laptop keyboard. In order to reflect the need for fast and accurate decisions within a match, participants were required to respond within 3 s of the completion of the clip, otherwise their response was recorded as incorrect. No performance feedback was given during the test. Referees taking part in this study were unfamiliar with videos seen during the test.

During the training phase, participants were required to judge the severity of foul situations while viewing clips shown on the same laptop screen. During training, participants viewed a total of 70 clips, which were taken from the Referee Assistance Programs 2015 and 2016 distributed by the Union of European Football Associations (UEFA). In advance of testing, participants were asked about their familiarity with the UEFA training program. All declared to be either unfamiliar with the program or admitted not to use the program despite their knowledge of existence of and/or access to the program. Only fouls were shown during training in order to expose participants to the type of kinematic actions that should characterize genuine fouls (Ryu et al., 2018). The task for participants during training was to choose as quickly and accurately as possible whether the situation warranted a red card, yellow card, or no card (foul only). The correct decision for each clip was provided in the program by UEFA, all according to Law 12 of the Game. In this way, the aim was for participants to be trained to better categorize fouls on the basis of the pick-up of the most essential information cues for these decisions. Participants performed three practice trials before the commencement of training. The clips were presented using the same randomized order for each participant. Each clip was preceded by the trial number and a countdown from three to one. Again, there was a time constraint of 3 s to make a decision and, unlike the pre-test, participants received direct feedback on their answer to encourage learning (Schweizer et al., 2011). After the first 35 clips, participants had the choice to take a small break or to continue with the training.

**FIGURE 1:**
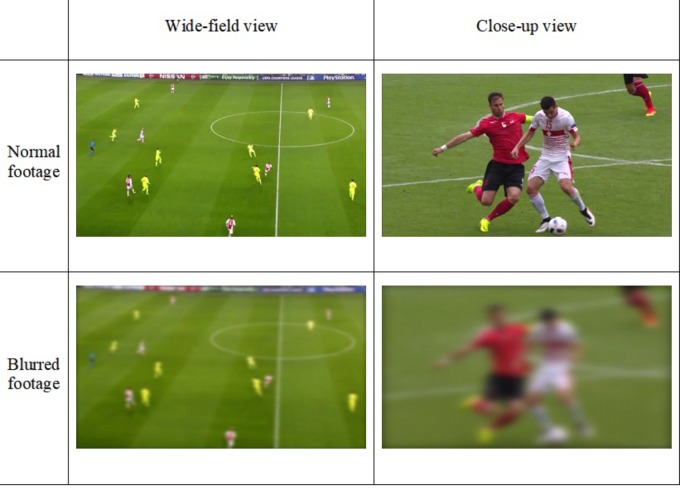
Demonstration of the amount of blur used in the training phase when compared to the standard view. Images are screenshots of the videos of the UEFA Referee Assistance Program used in the training. Permission of usage have been obtained by UEFA’s Referee Development Department.

Participants in the blurred-footage training group viewed video clips during training that were digitally altered using the camera blur option in Premier Pro CC software (Adobe Systems Incorporated, San Jose, CA, United States). Each video clip shown during training consisted of television footage that comprised a mixture of wide-field and close-up views of the situation. To achieve a relatively consistent level of blur within the clip, the wide-field parts of the clip were blurred using 7% blur, and the close-up parts of the clip were blurred with 20% blur (**Figure 1**). Those blur levels were chosen on the basis of mutual agreement between the authors during pilot testing where we compared blurred footage with blur used in previous studies (Mann et al., 2007; Bulson et al., 2008, 2015), with the overall aim to achieve a level of blur that would largely remove superficial visual information yet continue to make kinematic information available. Participants in the normal-footage training group viewed the original (unblurred) versions of the same video clips.

The procedure for the post-test was exactly the same as for the pre-test. In the post-test, each participant viewed video clips that they had not seen in the pre-test. The pre and post-test were counterbalanced in such a way that half of the participants viewed one set of 26 clips in the pre-test, and a different set of 26 clips in the post-test, while the other half of the participants viewed the two sets of clips in the opposite order.

### Data Analysis

Response accuracy was the key measure of decision-making performance. Response accuracy was scored on the pre-and post-tests by calculating the percentage of correct responses on each test. To test the hypothesis that blurred-footage training would result in a greater improvement in performance than control training, an independent *t*-test was used to compare the change in response accuracy from pre to post-test between the blurred-footage and normal-footage training groups. Follow-up paired *t*-tests were used to check whether there was a significant change in the performance of each group as a result of training, thus independently comparing the performance of each group to a null effect of zero. Moreover, *t*-testing was performed to check whether the response accuracy of the two groups differed in the pre-test.

We also calculated response time to ensure that any change in response accuracy was not a result of a trade-off between response accuracy and time. Response time was determined by calculating the time in milliseconds from the completion of the clip until the keyboard response was registered. An independent samples *t*-test was used to compare the change in response time from pre to post-test between the two groups to check whether response times changed as a result of training.

Signal detection analysis was used to check whether any changes in response accuracy following training could be attributable to changes in sensitivity or response bias (Cañal-Bruland and Schmidt, 2009; Bruce et al., 2012). This was particularly important because training consisted of only foul situations, so it was possible that participants could increase their bias to judge ambiguous foul situations as fouls following training. For signal detection analysis, responses were labeled as hits when participants correctly identified a foul (the “signal”), a miss when participants incorrectly judged a foul situation as no-foul, a correct-rejection when a no-foul clip was correctly judged as no-foul, and a false alarm when a no-foul clip was judged as a foul. To account for situations where the hit or false alarm rates could equal zero for a single participant, for the purposes of calculating hit and false alarm rates a log-linear approach was used (Stanislaw and Todorov, 1999), where 0.5 was added to each participant’s number of hits and false alarms, and one added to their number of signal and signal-absent trials. Sensitivity (d’) was defined as the ability to distinguish fouls from no-fouls and was calculated by subtracting the inverse of the standard normal cumulative distribution of the false alarm rate from that of the hit rate (Stanislaw and Todorov, 1999). Response bias (β) was defined as the tendency to favor either a foul or no-foul judgement, and was calculated by the formula *e*^0.5^∗^(z(FA)∧2-z(H)∧2)^, where FA is the false alarm rate, and H is the hit rate (Stanislaw and Todorov, 1999). Independent *t*-tests were used to check whether any change in the sensitivity and response bias as a result of training differed between the two groups. Data were tested for normality using the Shapiro–Wilk test; the Mann–Whitney U test was used instead of the *t*-test in any cases where the assumption of normality was violated. Effect sizes were calculated using Cohen’s d and expressed as a small (± 0.10), medium (± 0.30), or large effect (± 0.50) (Field, 2009). To evaluate the precision of the effect size, 95% confidence intervals were calculated for each effect size (95% CI_ES_) following the formulae of Nakagawa and Cuthill (2007). Alpha was set at 0.05 for all testing, with all analyses conducted using SPSS Statistics 22.

## Results

Tests for normality showed that all data for the control group were normally distributed, including the measures of response accuracy [pre-test; *D*(11) = 0.94, *p* = 0.56, post-test; *D*(11) = 0.86, *p* = 0.051, difference; *D*(11) = 0.92, *p* = 0.31], response time [pre-test; *D*(11) = 0.96, *p* = 0.70, post-test; *D*(11) = 0.93, *p* = 0.44, difference; *D*(11) = 0.92, *p* = 0.32], sensitivity [difference; *D*(11) = 0.95, *p* = 0.61] and bias [pre-test; *D*(11) = 0.96, *p* = 0.79, post-test; *D*(11) = 0.96, *p* = 0.82, difference; *D*(11) = 0.86, *p* = 0.05]. The data of the training group were normally distributed for response accuracy [pre-test; *D*(11) = 0.89, *p* = 0.10, post-test; *D*(11) = 0.90, *p* = 0.17, difference; *D*(11) = 0.88, *p* = 0.10], response time [pre-test; *D*(11) = 0.87, *p* = 0.07, post-test; *D*(11) = 0.85, *p* = 0.05, difference; *D*(11) = 0.92, *p* = 0.28] and bias [pre-test; *D*(11) = 0.86, *p* = 0.06, post-test; *D*(11) = 0.95, *p* = 0.61]. Therefore parametric testing was used for these variables. The data for the difference in sensitivity [*D*(11) = 0.85, *p* = 0.04] and the difference in bias [*D*(11) = 0.84, *p* = 0.03] from pre-to post-test were not normally distributed. For these variables, non-parametric Mann–Whitney U tests were used.

**FIGURE 2:**
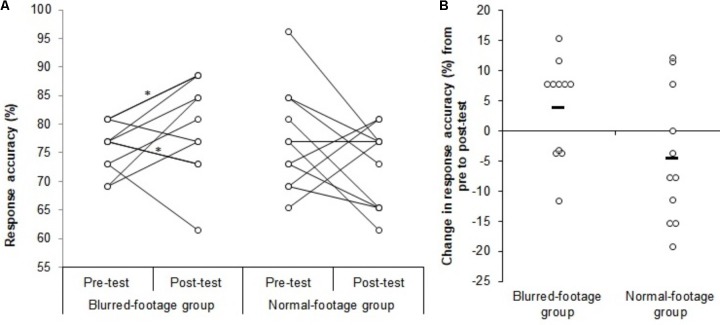
Change in decision-making performance from pre to post-test as a result of training for the blurred-footage and normal-footage training groups. Panel **A** shows each individual participant’s response accuracy in the pre and post-test, and panel **B** shows each individual’s *change* in response accuracy from pre to post-test. The black bars represent the means, the asterix (^∗^) indicates two participants having exact the same pre-test and post-test scores.

The blurred-footage training group’s change in response accuracy from pre to post-test was significantly greater than that for the normal-footage training group (**Figure 2**), planned *t*-test (one-tailed), *t*(20) = -1.012, *p* = 0.029, *β* = 0.487 [blurred-footage vs. normal-footage training group *(M ± SD)* = 3.9 ± 8.3% vs. -4.5 ± 11.0%], large effect size (Cohen’s *d* = 0.86), 95% CI_ES_ = 0.45–1.27. Follow-up *t*-tests showed that the blurred-footage training group experienced a borderline increase in response accuracy as a result of training, *t*(10) = -0.773, *p* = 0.07, *β* = 0.288 one-tailed [pre vs. post-test *(M ± SD)* = 75.9 ± 4.3% vs. 79.7 ± 8.5%], with a medium effect size (*d* = 0.47, 95% CI_ES_ = -0.26–1.20), whereas the normal-footage training group experienced a borderline decrease in performance, *t*(10) = 0.685, *p* = 0.10 one-tailed, *t*(10) = 1.370, *p* = 0.20, *β* = 0.237 two-tailed [pre vs. post-test *(M ± SD)* = 77.3 ± 8.9% vs. 72.7 ± 7.0%], with again a medium effect size (*d* = 0.41, 95% CI_ES_ = -0.42–1.24). There was no difference in response accuracy between the blurred-footage and normal-footage training groups at pre-test, *t*(20) = 0.473, *p* = 0.64, *d* = 0.20, 95% CI_ES_ = -0.18–0.58).

The difference between the two groups in the change in response accuracy following training could not be explained by changes in response times. A *t*-test comparing the change in response time from pre to post-test for the two groups showed that there was no significant difference between the blurred-footage and normal-footage training groups, *t*(20) = 0.617, *p* = 0.54, *β* = 0.090 two-tailed, *d* = 0.26, 95% CI_ES_ = -0.12–0.64 [blurred-footage vs. normal-footage training group *(M ± SD)* = -56 ± 126 ms vs. -4 ± 248 ms). Neither of the groups experienced a significant change in response time from pre to post test, blurred-footage group, *t*(10) = 1.462, *p* = 0.17 two-tailed, *d* = 0.44, 95% CI_ES_ = 0.16–0.72 [pre vs. post-test *(M ± SD)* = 842 ± 366 ms vs. 786 ± 356 ms], normal-footage group, *t*(10) = 0.051, *p* = 0.96 two-tailed, *d* = 0.02, 95% CI_ES_ = -0.44–0.48 [pre vs. post-test *(M ± SD)* = 833 ± 304 ms vs. 829 ± 327 ms).

**FIGURE 3:**
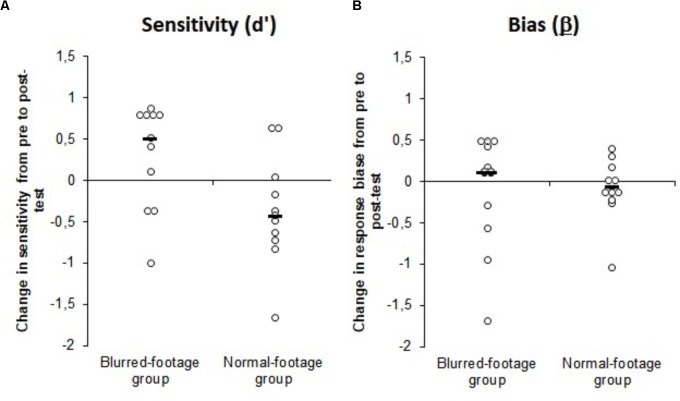
Change in **(A)** sensitivity and **(B)** response bias from pre to post-test as a result of training for the blurred-footage and normal-footage training groups. The black bars represent the means.

Signal detection analysis revealed that the differences in the behavior of the two groups could be explained by changes in their sensitivity to genuine fouls rather than a bias to expect fouls. A non-parametric Mann–Whitney test revealed a significant difference in the change in sensitivity for the two groups following training (**Figure 3A**), *U* = 25.00, *z* = -2.33, *p* = 0.02, *d* = 1.15, 95% CI_ES_ = 0.71–1.59 [blurred-footage vs. normal-footage training group *(M ± SD)* = 0.30 ± 0.63 vs. -0.37 ± 0.66]. This result showed that the ability to identify genuine fouls increased for the blurred-footage group when compared to the control (normal-footage) group. The analysis of response bias showed that there was no difference in the change in bias between the groups as a result of training (**Figure 3B**), *U* = 49.00, *z* = -0.76, *p* = 0.450, *d* = 0.33, 95% CI_ES_ = -0.05–0.71 [blurred-footage vs. normal footage group *(M ± SD)* = -0.12 ± 0.71 vs. -0.09 ± 0.38]. A response bias of β = 1 would indicate that referees favored neither a “foul” or “no-foul” call. The results showed that the response bias of the referees never varied significantly from β = 1 irrespective of the test, blurred-footage training pre-test, *t*(10) = -0.097, *p* = 0.92, *d* = 0.022, 95% CI_ES_ = -0.35–0.40 (*M* ±*SD* = 0.99 ± 0.45), blurred-footage training post-test, *t*(10) = -0.92, *p* = 0.38, *d* = 0.28, 95% CI_ES_ = -0.10–0.66 (*M* ±*SD* = 0.90 ± 0.36), normal-footage training pre-test, *t*(10) = 0.017, *p* = 0.87, *d* = 0.053, 95% CI_ES_ = -0.32–0.43 (*M* ± *SD* = 1.03 ± 0.57), normal-footage training post-test, *t*(10) = -0.57, *p* = 0.58, *d* = 0.18, 95% CI_ES_ = -0.20–0.56 (*M* ±*SD* = 0.91 ± 0.50). These results confirmed that the change in behavior of the groups following training cannot be explained by a change in any bias to favor a “foul” or “no foul” call.

## Discussion

The aim of this study was to determine whether perceptual training that removed superficial visual information would be effective for improving the decision-making performance of skilled football referees. When adjudicating ambiguous foul situations, referees must contend with the potentially deceptive actions of players who seek to fool them into awarding an unjustified foul. We hypothesized that blurred-footage training which removed superficial information would help referees to attune to the kinematic information associated with a genuine foul, leading to a significant improvement in decision-making performance. The results revealed that referees who performed a short period of training watching blurred footage experienced a significantly larger improvement in decision-making performance than referees who performed the same training without blur. The difference between the groups could not be explained on the basis of changes in response time or bias, but instead reflect a change in the sensitivity to genuine fouls. What is most remarkable is that the findings were uncovered in skilled referees, many of whom already perform at the highest level within their national competition. The findings suggest that the attunement to the putative kinematic information inherent within motor actions may hold promise as an effective means of improving the quality of decision making of officials in sport.

The results of this study are consistent with previous work which shows that the discrimination of deceptive from non-deceptive actions can be enhanced via attunement to the kinematic information inherent in an action sequence (Ryu et al., 2018). Previously, the suggestion has been that deceptive intent when performing motor actions is conveyed largely via very detailed non-kinematic information such as facial expressions and gaze direction (Abernethy et al., 2010a,b). In the case of a “dive” in football, it has instead been suggested that deceptive intent is largely conveyed via alterations in kinematics such as those resulting in the “archers bow” (Williams et al., 2006; Morris and Lewis, 2010; Lopes et al., 2014). Given that, in the case of adjudicating fouls, there are kinematic differences that characterize dives from fouls, then it stood to reason that blurred perceptual training could help referees to become more sensitive to the underlying kinematics and thereby to use that information to distinguish dives from fouls. The exposure to genuine fouls during blurred training may have helped referees attune to the kinematic signature that specifies when a player is fouled, helping the referees to better identify deceptive kinematic information when a player attempted a dive.

The decision whether an action is a foul or not is a very complex one (Johnson, 2006), with success in the task relying heavily on the visual and/or motor experience of the referee (Renden et al., 2014). Evidence demonstrates that perceptual training designed to improve the quality and/or volume of visual experience can positively contribute to the decision making performance of a referee (Catteeuw et al., 2010; Schweizer et al., 2011; Pizzera and Raab, 2012b; Put et al., 2013, 2016a,b). Therefore, we had a reasonable expectation that both of our training groups would improve their decision-making performance from pre to post-test. Evidently though, this was not the case. Specifically, the performance of the control group tended to decrease from pre to post test. Although this change fell short of significance (*p* = 0.10 using a conservative one-tailed *t*-test, *d* = 0.41 *β* = 0.237), the finding does justify further scrutiny. Given the wealth of previous studies which show video-based perceptual training to be an effective means of improving performance (Abernethy et al., 2012; Ryu et al., 2012; Put et al., 2013), it seems unlikely that the control video-based training in our study genuinely decreased the decision-making performance of the referees. Moreover, the inclusion of a corresponding control (e.g., placebo) group would have been necessary to disambiguate this conclusion from any effects of fatigue or boredom. The control training group was incorporated in our study to control for effects of learning and/or fatigue when evaluating the performance of the blurred-footage group, and it could be that the decrease in performance from pre to post-test can be better explained on the basis of fatigue and/or boredom. Fatigue does seem unlikely though given that our skilled referees are accustomed to long periods of decision making under cognitive and physical duress (a match lasts 90 min vs. 45 min for our procedure). Boredom could represent a viable explanation for our findings, particularly if participants in the control group experienced *more* boredom than those in the blurred-footage training group. Future work should look to include manipulation checks for cognitive engagement and/or boredom and to compare that across the intervention groups. To address another possible explanation, we applied signal detection analysis to determine whether any change in the type of responses made by the control group could be explained by a bias to judge a foul or not. Results revealed that there was no bias from either of the groups to judge a foul or not, and no significant difference in the change in the bias from pre to post-test between the two groups. Instead, the changes in response accuracy from pre to post-test were reflected by a change in the sensitivity to genuine fouls. The slight (non-significant) reduction in the response accuracy of the control group may be best attributed at this stage to chance. Follow-up and replication studies should reveal whether the slight decrease in performance for the control group can be replicated or is best attributed to chance.

Given, though, the poor post-test performance of the control group, it is important to consider whether the blurred-footage training group did experience their own change in decision-making performance as a result of training. The results show a ≈4% increase in decision-making performance for the blurred-footage training group, even after only approximately 20 mins of training (from 75.9 to 79.9%). On the basis of the planned expectation that the blurred-footage training group would improve their performance following training, we conducted a one-tailed *t*-test to test the significance of the change, with the effect falling just short of significance (*p* = 0.07, *β* = 0.288), though with a moderate measure of effect size (*d* = 0.47). These results do provide some tentative support for the potential efficacy of blur training as a means of improving the decision-making performance of football referees, though clearly further work is required to verify the findings, especially in the translation to on field decision making. Nonetheless to translate our findings, referees have been shown to make an average of 137 decisions per match, with approximately 60 of those related to foul situations (Helsen and Bultynck, 2004). An improvement of 4% would therefore equate to approximately 2.4 more correct foul-decisions per match. Since free kicks and penalties account for approximately 47.6% of all set play goals (∼25% of all goals) (Mitrotasios and Armatas, 2014), an increase in correct foul-decisions could have a significant impact on the outcome of a match.

A relatively unique aspect of this study is that training was conducted using skilled rather than novice participants. Most studies of perceptual training recruit novice participants (for an exception, see Hopwood et al., 2011), presumably to provide easy access to participants and to maximize the chance of finding a learning effect. The skilled referees who we tested in our study already possessed a high level of expertise in decision making, and so we ran the risk of not finding a training effect due to ceiling levels of performance (evidently this wasn’t a problem, with ≈75% pre-test response accuracy). Accordingly it is entirely possible that we have underestimated the size of any training effect when compared to most previous studies. Indeed the effect size we found for our blurred-footage training group (*d* = 0.47) is less than that found in comparable studies of perceptual training [*d* = 0.9–1.5; (Schweizer et al., 2011; Put et al., 2013; Ryu et al., 2018)], though those studies did typically test lesser-skilled rather than skilled participants and/or used longer periods of training.

It can of course be challenging to access a sufficient number of skilled participants for lengthy periods of time, and in this study we conducted the training over a period of time that was much shorter than that used in previous studies. The relatively brief access also meant that our participant numbers were limited (*n* = 11 per group); that we were not able to conduct a retention test of response accuracy as is customary in most training studies; and we did not conduct an on-field test of skill transfer. Clearly these improvements to the experimental design are desirable, and we hope that the relatively promising results found in this and other studies (e.g., Ryu et al., 2018) will lead to future work that addresses these shortcomings. In particular, future work should include a higher participant number to increase power and can look to establish what might be the optimal level of blur for training; the efficacy of longer training interventions ideally incorporating multiple training sessions; the retention of skill over a longer time-period; and the transfer of skill to check whether referees improve the quality of their on-field decision making (e.g., Put et al., 2013).

In the field of perceptual training in sport, there has been some general concern about the efficacy of training approaches which seek to train on-field skill using video-based stimuli (Starkes and Lindley, 1994; Dicks et al., 2015). In particular, there is concern that video-based training typically requires participants to produce verbal or button-press responses rather than producing genuine movement responses which replicate those that would typically be performed on-field (i.e., perception is decoupled from action). However, football refereeing represents a perceptual-cognitive task in which perception and action are largely decoupled, with referees required to make decisions which do not require a direct movement response. In this sense, video-based designs which decouple perception from action are more likely to be appropriate for testing and training referees than they might be for most athletes. In support, video-based training has previous been shown to improve the on-field performance of football referees (Put et al., 2013), providing confidence that video training is appropriate for our task.

## Conclusion

The results of this study show that blurred-footage training which removes superficial visual information may hold promise as a means of improving the ability of sport officials to discriminate “fouls” from “dives” when deciding whether to award a foul in ambiguous foul situations. It appears that the blurred intervention may have helped to train even skilled referees to attune to the kinematic information which characterizes a foul situation. Given Put et al.’s (2013) finding that web-based perceptual training can improve skill and enhance on-field decision making, the findings offer a potentially innovative means of enhancing the gains possible via perceptual training in refereeing. Future work should seek to establish whether there is an optimal level of blur for training along with whether a training schedule exists that maximizes benefits as a result of training.

## Data Availability Statement

The de-identified data are available from the corresponding author.

## Ethics Statement

This study was carried out in accordance with the recommendations of the national Code of Ethics for Research in the Social and Behavioural Sciences, The Scientific and Ethical Review Board (VCWE). The protocol was approved by the Vrije Universiteit Amsterdam Faculty of Human Movement Sciences Ethics committee (approval number VCWE-2016-212). All subjects gave written informed consent in accordance with the Declaration of Helsinki.

## Author Contributions

TvB, JK, and DM contributed to the conception and design of the study. TvB, PR, JK, and DM designed the experiments. TvB performed the measurements, processed the experimental data and wrote the first draft of the manuscript. TvB and DM and performed the statistical analyses. DM was involved in supervising the work and wrote sections of the manuscript. All authors contributed to manuscript revision, read and approved the submitted version.

## Conflict of Interest Statement

The authors declare that the research was conducted in the absence of any commercial or financial relationships that could be construed as a potential conflict of interest.
